# Implementing standardized criteria for multi-drug-resistant organisms: a retrospective cost-avoidance analysis for discontinuing contact precautions for ESBL

**DOI:** 10.1017/ash.2024.351

**Published:** 2024-09-26

**Authors:** Brenna Crossley, Carmen T. Cortes-Ramos, Dawn Nolt

**Affiliations:** 1 Infection Prevention and Control, Oregon Health and Science University, Portland, OR, USA; 2 Division of Infectious Diseases, Department of Pediatrics, Oregon Health and Science University, Portland, OR, USA

## Abstract

**Objective:**

This manuscript calculates the estimated cost-savings associated with implementing criteria for multi-drug-resistant organisms (MDRO).

**Design:**

The study evaluated extended-spectrum beta-lactamase (ESBL) producing *Enterobacterales* isolates utilizing the MDRO criteria established by Infection Prevention and Control. Isolates were categorized as either meeting or not meeting criteria. The number of inpatient days for patients with isolates not meeting criteria was calculated. The average daily cost of personal protective equipment (PPE) for patients in contact isolation was determined via literature review. Annual cost savings were determined by multiplying the total number of inpatient days by the average cost of PPE per day. Because our institution only isolates patients who meet the MDRO criteria, this approach was considered a cost-saving measure.

**Setting:**

560 licensed bed, tertiary care facility in the United States.

**Patients:**

Adult inpatients between the years of 2019–2022 with an ESBL-producing *Enterobacterales* isolated from any specimen source.

**Results:**

229 patients met inclusion criteria. 73% of isolates did not meet MDRO criteria. The patients with ESBL isolates not meeting criteria represented 2942 isolation days over four years. The average cost of PPE for contact isolation per day was $40.18. Cost-savings were estimated at $118,209 over four years.

**Conclusions:**

Our findings provide support for other healthcare systems to define organisms that warrant transmission-based contact precautions.

## Introduction

Antimicrobial resistance continues to be an urgent public health threat. Almost 3 million antimicrobial infections occur in the United States annually and are associated with significant morbidity and mortality.^1^ Multi-drug resistance can occur through many different mechanisms, but the outcome is similar: less available antibiotics to treat infections.

There is no universal definition of multi-drug-resistant gram-negative bacilli (MDR-GNB); however, extended-spectrum beta lactamase (ESBL) is a commonly accepted definition for gram-negative bacteria that shows a constellation of resistance patterns to cephalosporins, penicillins, and aztreonam. Transmission-based precautions, particularly contact precautions, are a common infection prevention and control intervention to prevent the spread of multi-drug-resistant organisms (MDROs) in healthcare settings.^2^

Out of an abundance of caution and due to lack of a standardized definition for MDR-GNB, many healthcare institutions place patients infected or colonized with ESBL-producing organisms into contact precautions. While contact precautions may safeguard against direct and indirect transmission of MDROs within a healthcare facility, unnecessarily isolating patients can be a significant burden to the healthcare worker, patients, and the healthcare institution.^3,4^ These burdens include decreased medical interventions, reduced patient satisfaction, increased waste of personal protective equipment (PPE), and higher healthcare costs. To mitigate undesirable impacts of contact precautions, in 2019 our healthcare system implemented standardized criteria for MDR-GNB utilizing the definition of extensively-drug-resistant and pan-drug-resistant organisms developed by Magiorakos et al.^5^ In short, our facility targeted isolation for patients colonized or infected with organisms having fewer than three classes of antibiotics available for treatment.

This manuscript quantifies the PPE cost savings of isolating only patients colonized or infected with institutionally defined MDROs compared to isolating all patients with an ESBL-producing *Enterobacterales*, as is the common protocol for many institutions.

## Methods

Data was collected retrospectively from a 560 licensed bed, tertiary care facility in the United States. The facility is comprised of 330 adult non-ICU bed, 80 adult ICU beds, 130 pediatric non-ICU beds, and 20 pediatric ICU beds. The facility has 26 double-occupancy rooms (all within the adult non-ICU units). The facility utilizes Epic Systems (Verona, Wisconsin) as the electronic health record system and TheraDoc (Salt Lake City, Utah) as the surveillance software. Per institutional policy, patients with MDRO colonization or infection are placed in contact isolation for 1 year following the date of the most recent specimen collection containing the MDRO. The facility’s institutional review board waived oversight for this project.

ESBL-producing *Enterobacterales* isolates collected from adult inpatient units between January 1, 2019–December 31, 2022 were extracted from the surveillance system. The facility utilized multiple microbiology laboratories during the study period; however, all laboratories are accredited by the College of Amerian Pathologists, and utilize the breakpoints established by the Clinical and Laboratory Standards Institute to determine antimicrobial susceptibilities. A combination of susceptibility patterns and ESBL confirmatory testing were used to identify isolates. Isolates collected in ambulatory areas and the emergency department were not included in the study; patients <18 years of age were also excluded.

Because hospitalized patients may have multiple cultures collected during a single hospital admission, results were deduplicated by Medical Record Number to account for each patient only once per admission. After exclusions, 229 isolates remained. Included isolates were evaluated against the MDRO criteria utilized by the facility’s Infection Prevention and Control Department (Appendix). Isolates were separated into two categories: “Meets MDRO criteria” and “Does not meet MDRO criteria.”

To calculate the financial impact of differential isolation, the number of isolation days were calculated for the patients included in the “Does not meet MDRO criteria” category. Admission data was abstracted from the surveillance system. Isolation days were calculated by counting the number of days from the first ESBL isolate collected from the inpatient location to the date of discharge. For example, if an isolate was collected halfway through a patient’s admission, only the last half of that admission was counted towards the isolation days calculation. Subsequent admissions within one year of the ESBL isolate collection date were included in the isolation days calculation. Admissions were excluded from calculation if any of the following transpired: patient admitted prior to the collection of the ESBL isolate or occurred greater than 1 year from the collection of the ESBL isolate.

Direct cost savings from PPE use associated with discontinuing contact precautions for ESBL patients were calculated by multiplying the cost of PPE per day, derived by Saber et al.^6^ by the total number of isolation days for patients in the “Does not meet MDRO criteria” category.

## Results

Sources of isolates: Of the 229 ESBL isolates, 167 (73%) did not meet internal criteria for MDRO. Of the subset that did not meet MDRO criteria, 87 were from urine (52%), 21 were from blood (13%), 10 were from respiratory sources (6%), and 49 were wounds or other (29%). There were 62 isolates (27%) that met internal criteria for MDRO, meaning the isolate was non-susceptible to at least one agent in all but 2 or fewer antibiotic classes (Appendix). Urine accounted for the greatest number of MDRO isolates (*n* = 26, 42%), followed by wound or other (*n* = 19, 31%) and bloods (*n* = 16, 26%). There was one MDRO isolate collected from a respiratory specimen. There was a numerical difference in the percentage of MDROs isolated from blood compared to all ESBL, but this did not meet statistical significance (*P*-value = 0.41) per chi-squared test of independence.

Cost avoidance (Table 1): The average direct cost of PPE (gowns and gloves) associated with contact precautions was derived from Saber et al, and was estimated in 2022 at $40.18 USD per patient day.^6^

**Table 1.** Cost estimates for isolating patients with ESBL which do not meet institutional MDRO criteria

	2019	2020	2021	2022	Total
Number of patients	32	30	33	46	**141**
Number of isolation days	539	780	605	1018	**2864**
Cost in dollars (Number of isolation days * PPE^a^)	$21,657	$31,340	$24,308	$40,903	**$118,209**

a Derived from Saber et al. at $40.18/day for contact isolation.^6^

Patients in the “Does not meet MDRO criteria” category represented 2942 isolation days over four years. Cost avoidance is estimated at $118,209 over four years.

Balance measures: Annual institutional antibiograms did not exhibit changes in gram negative susceptibilities (Table 2). No outbreaks of ESBL organisms were seen in the same period. Review of hand hygiene observation data showed >95% compliance for the years included in the study. There was no difference in compliance pre and post-policy change. Environmental cleaning chemicals were not changed during the study period.

**Table 2.** Percentage of *Enterobacterales* clinical isolates found to be ESBL producing (2016–2022), inferred from ceftriaxone susceptibility

	*Escherichia coli*	*Klebsiella pneumoniae*	*Proteus mirabilis*
2016	90	93	100
2017	90	91	97
2018	91	95	98
2019	92	95	97
2020	90	95	99
2021	95	96	99
2022	90	92	98

## Discussion

Implementing standardized criteria for MDRO classification can be a cost-saving to the institution. By discontinuing the requirement to place patients with an ESBL into contact precautions, the institution saved approximately $118,000 in direct PPE costs over the four-year study period, without increased prevalence or outbreaks of resistant gram-negative organisms. While this paper only evaluated adult inpatients, the potential savings from using MDRO criteria for initiation of contact precautions could also be extrapolated to the emergency department, pediatrics areas, and ambulatory clinics.

Although classifying bacteria as ESBL is one way to designate drug-resistant pathogens, it is not the only method. The paper by Magiorakos was originally designed to standardize the definition of MDRO for research purposes. Although it was not intended to be a framework for transmission-based precautions, our institution utilized that publication as one method to determine “difficult-to-treat” organisms, which would be undesirable to disseminate in the healthcare setting.

In the same way, there is no universal definition for MDRO, there is also no universal isolation recommendation for these organisms. Centers for Disease Control and Prevention (CDC) Appendix A provides guidance for type and duration of transmission-based precautions for selected infections and organisms.^7^ For MDROs, CDC recommends utilizing a multitude of resources for determining which organisms are clinically and epidemiologically significant for individual institutions. An informal inquiry into regional healthcare facilities through the Association for Professionals in Infection Control and Epidemiology (APIC) showed that isolation policies for ESBL are varied. While this paper utilized 1 year as the basis for isolation due to institutional policy, not all organizations utilize that specific duration. Our cost-savings may be over or under-represented depending on local policy, including the utilization of screening tests and shorter durations of isolation. Additionally, it would be paramount for institutions (such as skilled nursing facilities and long-term care facilities) to perform an individual risk assessment to determine the implications of discontinuing contact precautions on inter-facility transmission.

Introduction of new antimicrobial categories with accompanying new agents can always allow organisms to fulfill criteria for susceptibility, and will eventually render the classifications by Magiorakos as unsustainable. There will be a need to continually define the MDRO definition over time as more information is gained regarding new antibacterial agents and new pathways of antimicrobial resistance.^8^ There is not an anticipated update to the Magiorakos publication (Arjun Srinivasan, personal communication).

Several assumptions were used in the financial calculations. First, attempts to calculate institution-specific material costs were difficult as units of PPE ordered did not necessarily correlate with units of PPE utilized for patients in contact precautions. This discrepancy led the authors to utilize a fixed PPE price from a direct observational study^6^, which would be more accurate than calculations utilizing purchasing data. Second, the fixed PPE cost was determined in 2022 and did not account for price fluctuations, particularly in times of shortages during the COVID-19 pandemic.^9^ Additionally, our findings only account for direct material costs of PPE. Saber et al. referenced additional costs, including direct labor costs (i.e. staff time to don and doff PPE, cleaning by environmental services) and indirect costs (i.e. sourcing, receiving, and storage of PPE). These costs are not included in this study due to vast differences among facilities. We assumed that all patients had equivalent medical acuity and complexity, though those with higher acuity and/or complexity would necessitate more personnel interactions and thus more PPE use and cost.

This study has several limitations. It is a single-center study at a tertiary healthcare facility, which precludes extrapolation to other healthcare settings that may not possess patients with MDRO and thus would not exhibit the same degree of cost savings. Most of the observations occurred during the COVID-19 pandemic, which skewed the hospitalized patient mix and thus may have affected the number of inpatient individuals who were colonized and isolated for ESBL/MDRO. Although no obvious clinical harm was determined (based on lack of recorded outbreaks), concurrent clinical surveillance was not performed, which may have provided evidence of any direct patient-specific harm.

Creating standardized criteria for MDRO ensures that patients are not over-isolated without obvious dissemination of resistant organisms. Our institutional findings and literature support that discontinuing contact isolation for ESBL does not increase transmission of these organisms and highlights the importance of standard precautions for prevention.^10,11^ This study shows the positive financial impact of an institutional workflow in utilizing contact isolation for selected hospitalized adult inpatients with MDRO.
